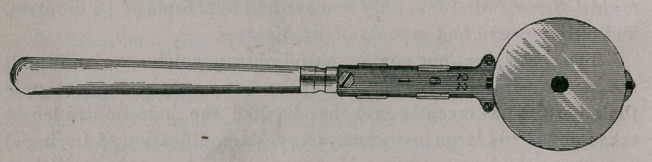# Philadelphia County Medical Society

**Published:** 1891-11

**Authors:** 


### Society Proceedings.

### PHILADELPHIA COUNTY MEDICAL SOCIETY.

*Stated Meeting, September* 9, 1891.

The President, John B. Roberts, M. D., in the Chair.

Dr. Edward Jackson read a paper on

### AN OPHTHALMOSCOPE FOR GENERAL USE.

It would be a great gain to both doctors and patients if a much larger proportion of those who class themselves as general practitioners were able, when the need for it arose, to use the ophthalmoscope. One who has no practical experience with it cannot even properly appreciate what he reads or hears of ophthalmoscopic appearances. And there are in the aggregate many cases in which the progress of general disease could be far more intelligently followed by its routine use, without entering upon debatable ground or attempting to use symptoms of doubtful significance.

With the ophthalmoscope, as with other instruments, the cheap instrument is very apt to lack certain important features, and the costly instrument is mainly confined to the possession of those who mean to use it a good deal. It took many years to adapt the microscope to the needs of clinical work, to rid it of mechanical stages and other mechanical nuisances, and perfect its really essential parts. And the ophthalmoscope must pass through a similar pruning and adaptation before its use can be truly popular and common in the profession. For some years I have been working at this problem, and herewith present my results.

The ophthalmoscope for general use must: First, be one in which the difficulties of using the instrument are, as far as possible, overcome. Second, it must be one that will be as satisfactory as any of the best instruments for any case that is likely to be encountered. Third, it must be cheap. For this one I have no hesitation in claiming that with it the fundus of the eye can be seen as readily as with any ophthalmoscope heretofore made; for all practical purposes as a refraction ophthalmoscope, its lens series is complete; it can be bought for eight dollars.

It is easy to see through, because the mirror, which is circular, 30 mm. in diameter, tilts each way to the best angle, at about 25 or 30 degrees; it has a shorter canal and wider lenses than have most first-class refraction ophthalmoscopes; each lens is retained in exact position by a spring stop; and all the lenses or combinations of lenses are available without taking the instrument from the eye.

The lens series is furnished by combinations of six lenses in two slides, and consists of convex 1, 2, 3, 4, 6, and 12 dioptres; concave 1, 2, 4, 6, 10, and 22 dioptres. To appreciate this series, one must bear in mind the degrees of ametropia that are commonly encountered in practice. Among 4,000 eyes, the statistics of which I have published in the *Transactions of the American Ophthalmological Society* for 1889, only one eye had hyperopia of 13 dioptres, and only one eye had myopia of 23 dioptres.

The series does not contain half-dioptres, which are given in all the larger refraction ophthalmoscopes; but a very prominent ophthalmologist has recently said that he had the half-dioptre lenses taken out of his large instrument (Noyes's modification of Loring's) as comparatively worthless. Under especially favorable conditions there are a few ophthalmoscopists who have constant and extensive practice with the instrument who can, I believe, measure refraction with a little more exactness with half-dioptre lenses than they could with only whole-dioptre intervals. But the ophthalmoscopists that can do this are comparatively few, the cases in which they can do it are few, and the practical value of doing it is utterly insignificant. For those who are not in special practice half-dioptre intervals are always a delusion and snare, a hindrance, a cause of inaccuracy. They are, therefore, discarded.

Although the statistics above referred to show that in but one eye in forty of those encountered in practice is the degree of ametropia over 6 dioptres, to one not very familiar with the properties of lenses the intervals between the stronger lenses of this series may seem too great. Such must be reminded that the effect of every intermediate lens strength may be obtained by slightly varying the distance of the lens and instrument from the patient's eye. Thus the convex 6-dioptre lens acts as such only when placed against the eye; by drawing it back less than three inches it is made to act as a 12-dioptre lens, and within that space will correct any intermediate amount of hyperopia. By withdrawing the 12-dioptre convex lens a little over one inch, it takes the place of a 20-dioptre lens. On the other hand, by withdrawing the concave 22-dioptre lens a little over two inches, its effect is diminished to 10 dioptres, and in that space every intermediate strength is reproduced. In the same way, the withdrawal of the 10-dioptre concave lens to the same distance gives us the 6-dioptre effect.

When this is remembered, it is readily seen that any measurement of refraction by strong lenses is utterly untrustworthy unless the distance of the lens from the eye is taken into account; and if it is taken into account, any additional intermediate lenses are quite unnecessary. The above series is sufficient for the direct method in all cases except the very highest myopia, for which the expert ophthalmoscopist is apt to resort to the indirect method as more satisfactory.

To one accustomed to using a disc ophthalmoscope, the arrangement of lenses as here, in slides, will at first seem awkward and confusing, but to one who begins with this instrument, or who has already used an instrument in which the lenses are so placed, it is especially convenient. The convex lenses are all in the back slide, the concaves in the front. One can be used alone, or both slides can be moved at once by the tip of the same forefinger, according to the lens required.

In the focus of the mirror, the size of the sight hole, the blacking of it, the proportioning of the instrument, and its mechanical execution, it is equal to the best ophthalmoscopes now used. It is made by Mr. D. V. Brown, of Philadelphia.

Since this is not my first attempt at the modification of the ophthalmoscope, and another instrument has my name associated with it, perhaps it will prevent confusion if I exercise the right of naming this. And with the idea of giving it a name that shall by a single word indicate the idea of its design for general use, I shall call it the Polyclinic Ophthalmoscope.

## Figures and Tables

**Figure f1:**